# Sasang constitution affects the prevalence of functional dyspepsia

**DOI:** 10.1186/s12906-015-0674-8

**Published:** 2015-05-20

**Authors:** Yoon Jeong Kim, Yo Chan Ahn, Chang Gue Son

**Affiliations:** Liver and Immunology Research Center, Daejeon Oriental Hospital of Daejeon University, # 176-9 Daeheung-ro, Jung-gu, Daejeon, 302-724 Republic of Korea; Department of Health Service Management, Daejeon University, # 62 Daehak-ro Dong-gu, Daejeon, 300-716 Republic of Korea

**Keywords:** Functional dyspepsia, Sasang constitution, Traditional Chinese medicine, Traditional Korean medicine

## Abstract

**Background:**

Functional dyspepsia (FD), which is a very common disorder worldwide, is known to be caused by multiple factors including environmental and genetic factors. Sasang constitutional medicine (SCM) is a component of traditional Korean medicine that emphasizes inherited characteristics of the physical and psychological patterns of a patient. This study investigated whether the prevalence of FD differs depending on Sasang classification.

**Methods:**

A total 517 subjects (190 males and 327 females) were recruited, and interviewed for the presence of FD using a Rome III-based questionnaire. The Sasang constitution of all subjects were diagnosed using a Sasang constitutional analytical tool (SCAT). A Chi-square test was performed to compare prevalence of DF among different Sasang constitutional types.

**Results:**

Of the 517 subjects, 115 (22.2 %) met the diagnostic criteria for FD, and the prevalence was significantly higher in females (26.9 %) than males (14.2 %, *p* < 0.01). The Sasang-constitution-based prevalence among all subjects was 27.5 % for Taeumin, 23.1 % for Soumin, and 16.4 % for Soyangin (*p* = 0.055). When compared by sex, the prevalence of FD among Sasang types showed significantly different patterns between males and females (*p* < 0.05); in females with FD, Taeumin predominated (32.5 % compared with 29.5 % and 18.8 % for Soumin and Soyangin, respectively; *p* < 0.05), whereas males with FD displayed a higher prevalence of Soumin (17.3 % compared with 9.1 % and 11.3 % for Taeumin and Soyangin, respectively; *p* > 0.05).

**Conclusions:**

This study identified significant differences in FD prevalence depending on Sasang constitution and sex. Our findings provide data to guide future research on the prevention and management of FD.

## Background

Functional dyspepsia (FD) is defined as a condition in which dyspeptic symptoms occur in the epigastric region in the absence of an organic disease that explains them [1]. FD is one of the most common medical conditions, with a worldwide prevalence of approximately 11–29.2 % [2]. Symptoms of FD are non-specific, and the disorder seriously impairs patients’ daily functioning and quality of life [3].

The pathophysiology of FD is highly diverse, and involves delayed gastric emptying, hypersensitivity to gastric distension, altered gastrointestinal motility and gastric electrical rhythms, and dysregulation of the autonomic nervous–central nervous system relationship [4, 5]. The main pathogenetic factors of FD include genetic predisposition, psychosocial factors, infection from *Helicobacter pylori*, and inflammation [6, 7]. These multicausal and multifactorial characteristics of FD partially explain why few universally effective therapies exist for the disorder [8]. Accordingly, patients with FD frequently use complementary and alternative medicine, including herbal remedies and acupuncture, as an important choice alongside conventional treatment [9, 10].

Sasang constitutional medicine (SCM), a field of traditional Korean medicine (TKM), emphasizes the inherited characteristics of psychological and physical patterns, as well as the combination of congenital determinants and internal organ function in the development of diseases or disorders [11]. Based on these inherited traits, SCM classifies people into four types: Taeyangin, Soyangin, Taeumin, and Soumin [12]. Several studies have proposed constitutional-type-specific susceptibility to certain disorders, such as a high prevalence of ischemic stroke in Taeumin and lower digestive function in Soumin subjects [13, 14]. Additionally, a genetic role in the development of FD has been well demonstrated [15, 16]. However, few studies have investigated the relationships between the development of FD and Sasang constitution classification.

The present study investigated how FD prevalence varies according to Sasang constitution.

## Methods

### Subjects

In total, 517 participants (aged 17**–**77 years) were recruited among general population in Daejeon city of South Korea, from November, 1^st^, 2013 to December, 31st, 2014 (Table 1). Participants consisted of 190 males and 327 females, and their occupations were varied like college student, office worker, and others. Any subject who had to take a medicine due to a distinct organic disease was excluded from the study. Informed consent was obtained from each subject, and the Ethics Committee of Daejeon University Hospital approved the study protocol (Authorization number: DJOMC-118).

**Table 1:** Characteristics of study participants

Characteristics	Male	Female	Total
Number (%)	190 (36.8)	327 (43.2)	517 (100)
Median age (years, range)	23 (18–64)	21 (17–77)	21 (17–77)
Median height (cm, range)	173.5 (161.3–189.2)	161.0 (142.6–176.0)	164.8 (142–189.2)
Median weight (kg, range)	70.2 (44.3–96.6)	55.8 (40.4–98.0)	60.7 (40.4–98.0)
Mean BMI ± SD	23.5 ± 2.8	21.9 ± 3.5	22.5 ± 3.3

### Diagnosis of FD

Based on a self-reported questionnaire, potential participants were interviewed by a doctor to ascertain the presence of FD according to the Rome III Diagnostic Criteria [17], which are: patient complains of one or more of bothersome postprandial fullness, early satiation, epigastric pain, or epigastric burning lasting at least 3 months with symptom onset at least 3 months prior to diagnosis. Subjects diagnosed with FD did not have any structural disease that was likely to explain their symptoms, as determined by a doctor’s interview regarding whether an endoscopy had been performed, physical examination, laboratory (hematological and biochemical) tests, and medical history.

### Classification of Sasang constitution

Each participant was classified as having one of the four types of Sasang constitution (Taeumin, Taeyangin, Soyangin, or Soumin) using the Sasang constitutional analytical tool (SCAT). The SCAT is a web-based Sasang constitution decision-making system that was developed by the Korea Institute of Oriental Medicine [18]. The selection of Sasang constitution is made by an integrative combination of information from the subject’s body shape (10 points), facial contour analysis (10 points), voice features (5 points), along with physiologic symptoms collected by questionnaire (10 points). Once individual levels of all required items were entered into the SCAT system, it presented the percentage of each person's potential to be categorized as a Sasang constitution type. Then, a person's Sasang constitution with the highest percentage is adopted as the person's final type.

### Statistical analysis

Statistical analysis of the data was performed using SAS statistical software (SAS Rel. 8.02; SAS Institute, Inc., Cary, NC, USA). A Pearson’s Chi-square test or two-way ANOVA was used to compare the prevalence of FD in terms of Sasang constitution type and sex. *P*-values <0.05 were considered statistically significant.

## Results

### Prevalence of FD by sex

Among 517 participants, 115 met the diagnostic criteria for FD, for a total prevalence of 22.2 %. The prevalence in males was 14.2 % (27 of 190 subjects); it was significantly higher in females, at 26.9 % (88 of 327 subjects, *p* < 0.01, Table 2).

**Table 2:** Distribution of patients with FD according to Sasang classification and sex

Subjects (%)		Taeumin	Soumin	Soyangin
Total subjects	Male: 190 (100)	33 (17.4)	104 (54.7)	53 (27.9)
	Female: 327 (100)	120 (36.7)	95 (29.1)	112 (34.3)
	Total: 517 (100)	153 (29.6)	199 (38.5)	165 (31.9)
Subjects with FD	Male: 27 (100)	3 (11.1)	18 (66.7)	6 (22.2)
	Female: 88 (100)	39 (44.3)	28 (31.8)	21 (23.9)
	Total: 115 (100)	42 (36.5)	46 (40.0)	27 (23.5)
FD prevalence ^**, #^	^α^Male: 14.2 %	9.1 %	17.3 %	11.3 %
	^*^Female: 26.9 %	32.5 %	29.5 %	18.8 %
	Total: 22.2 %	27.5 %	23.1 %	16.4 %

A comparison of FD prevalence between males and females (^**^ *p* < 0.01) and among Sasang constitution types in male (^α^ *p* = 0.389), female (^*^ *p* < 0.05), and all subjects (^β^ *p* = 0.055) obtained by Pearson’s Chi-square test. The sex-dependent difference in FD prevalence in terms of Sasang constitution (^#^ *p* < 0.05) was shown via a two-way ANOVA test

### Distribution of Sasang constitutions

The proportions of Taeumin, Soumin, and Soyangin constitution types among the total participants were 29.6 %, 38.5 %, and 31.9 %, respectively. When the Sasang constitution was analyzed according to sex, male subjects showed prevalence of 17.4 %, 54.7 %, and 27.9 %, and female subjects showed prevalence of 36.7 %, 29.1 %, and 34.3 % for Taeumin, Soumin, and Soyangin, respectively. Among the 115 subjects with FD, the Sasang distribution was 36.5 % (11.1 % in males, 44.3 % in females), 40.0 % (66.7 % in males, 31.8 % in females), and 23.5 % (22.2 % in males, 23.9 % in females) for Taeumin, Soumin, and Soyangin, respectively (Table 2).

### Prevalence of FD among Sasang constitutions

The differential distribution of FD prevalence according to Sasang constitution type was: 27.5 % for Taeumin, 23.1 % for Soumin, and 16.4 % for Soyangin (*p* = 0.055). When analyzed separately by sex, the prevalence of FD according to Sasang constitution differed significantly according to sex. More women FD prevalence was of the Taeumin type (32.5 % compared with 29.5 % and 18.8 % for Soumin and Soyangin, respectively, *p* < 0.05). The prevalence of FD among men did not differ significantly according to Sasang type (*p* = 0.389), although there was a Soumin-dominant tendency (17.3 % compared with 9.1 % and 11.3 % for Taeumin and Soyangin, respectively). This sex-dependent difference in the pattern of FD prevalence was significant (*p* < 0.05, Table 2).

## Discussion

Current genetic science has shown that inherited genomic variance is a critical factor affecting individual differences in the development of various diseases [19]. Genomic or constitutional components are closely related to the occurrence of FD [20, 21]. SCM emphasizes the importance of the inherited characteristics of psychological and physical patterns in the development of disease and in therapeutic responses [22]. Accordingly, it is assumed that each type of Sasang constitution shows a different susceptibility to FD.

Our results reveal that 22.2 % of the total subject was belonged in FD. This result is similar to the data from a Korean nationwide survey showing a 20.4 % prevalence [23]. The prevalence of FD has varied considerably depending on the population or definition used. The 14.7 %, 17 %, and 23.8 % prevalence were reported from studies in Norway [24], Japan [25], and the US [26], respectively. The presence of true FD, where organic disease is excluded, is difficult to determine in population studies due to logistic difficulties [2].

SCM classifies people into four constitutional types based on the weighted *Yin-Yang* balance and the functional imbalance between two internal organs; Taeyangin (greater *Yang*) individuals are characterized as having excessive lung function and deficient liver function, whereas Taeumin (greater *Yin*) individuals show the opposite pattern. Soyangin (lesser *Yang*) individuals have excessive spleen function and deficient kidney function, whereas Soumin (lesser *Yin*) individuals show the opposite pattern [27]. It is generally understood that the Taeyangin type is extremely rare in the Korean population [28], and none of the 517 study subjects in the present study was classified as Taeyangin.

Despite a general belief that the prevalence of FD is relatively equal in males and females, two studies, from Taiwan and Australia, showed significantly higher FD prevalence in females compared to males [29, 30]. Both the previous Korean study [23] and the present study found a higher prevalence of FD in females by approximately 2-folds (14.2 % of male *versus* 26.9 % in females). When the FD prevalence was investigated according to Sasang constitution, Taeumin (27.5 %) and Soumin (23.1 %) individuals displayed notably higher FD prevalence compared with Soyangin (16.4 %) subjects, a difference that approached statistical significance (*p* = 0.055). Our results also found especially a Sasang-type-dependent difference in susceptibility to FD, even within the same sex. We observed a Taeumin-prevalent pattern in females *versus* a Soumin-prevalent pattern in males. Our data may suppose the highest susceptibility of FD in Taeumin female *versus* the lowest FD susceptibility in Taeumin male, as 3.6-fold higher in male comparing to female. This finding may indicate the different manners of affections by Sasang constitutional characteristics between males and females. Sex-related differences in the development of various diseases are well known [31]. Our previous study found a significant difference in cancer prevalence according to Sasang classification and gender, with higher cancer prevalence in Soumin-type males and Soyangin-type females [32]. These findings would recommend the Sasang constitution as an important medicine in diagnosis or therapeutics of FD.

Recently, several studies have reported that subjects of certain Sasang classifications are notably more susceptible to some disorders; e.g., Taeumin for type 2 diabetes and metabolic syndrome [33, 34] and Soumin for irritable bowel syndrome [35]. We are unable to explain the mechanisms responsible for the association between Sasang constitution and the tendency to develop particular disorders, including FD. However, there are some clues to support the clinical phenomena. SCM classification is assumed to be connected to macro-level genomic differences [36, 37]. Biological and psychological characteristics including diet habits are associated with Sasang constitutional types [38, 39]. Interestingly, one study proposed a possible link between Sasang type and gut microbial environment [40]. Psychological characteristics, dietary habits, and gut flora are all known as factors that strongly affect the development of FD [41].

FD is a very common disorder that reduces health-related quality of life and impairs vitality. However, currently, no standard effective therapy exists due to the multicausal and multifactorial characteristics of the disorder [42]. Our findings demonstrate firstly an association between Sasang constitution and FD prevalence. Our study however had limitations, such as the relatively small number of subjects from a single city in Korea. We therefore have to pay careful attention in interpretation of our finding, which should be confirmed by a large scale of study in the future. We also need the further studies explaining the corresponding mechanisms of differences in FD prevalence among Sasang constitutional types, for example regarding any patterns of genomic differences, psychological characteristics, diet habits, and gut microbial environment.

## Conclusion

In conclusion, our results identified a significantly different pattern in FD prevalence among Sasang constitutions, and this pattern differed by sex. These findings will benefit future research on the prevention and management of FD.
